# Impact of Feeding Cover Crop Forage Containing Brassicas to Steers during Backgrounding on Palatability Attributes of Beef Strip Steaks

**DOI:** 10.3390/foods10061250

**Published:** 2021-05-31

**Authors:** Christina Bakker, Lydia Hite, Cody Wright, Alexander Smart, Thu Dinh, Amanda Blair, Keith Underwood, J. Kyle Grubbs

**Affiliations:** 1 Department of Animal Science, South Dakota State University, Brookings, SD 57007, USA; Christina.Bakker@sdstate.edu (C.B.); Lydia.hite@sdstate.edu (L.H.); Cody.Wright@sdstate.edu (C.W.); amanda.blair@sdstate.edu (A.B.); keith.underwood@sdstate.edu (K.U.); 2 Department of Natural Resource Management, South Dakota State University, Brookings, SD 57007, USA; Alexander.Smart@sdstate.edu; 3 Department of Animal and Dairy Sciences, Mississippi State University, Starkville, MS 39762, USA; thu.dinh@msstate.edu

**Keywords:** beef, brassica, proteolysis, tenderness, turnip, radish

## Abstract

Brassica cover crops have been widely used for improving soil health and as a feed resource for grazing cows, but their use in backgrounding diets is unknown. The objective of this study was to determine the impact of feeding a brassica cover crop mixture during backgrounding on beef palatability. Thirty steers were assigned to one of two dietary treatments during backgrounding with (1) ad libitum access to freshly cut brassica cover crop forage (CC) containing radish, turnip, rapeseed, and rye grass, or (2) common Midwestern dry lot backgrounding diet (CON). The steers were transitioned to a common finishing diet after backgrounding. Striploins were collected after harvest, and were analyzed for evaluation of the Warner–Bratzler shear force (WBSF), collagen content, autolysis of calpain-1, proteolysis of desmin, and troponin-T; in addition, the tenderness, juiciness, and flavor evaluated by a trained sensory panel. A treatment x day interaction was observed for WBSF (*p* = 0.02). Steaks from the CON diet were less tender than CC steaks on days 3 and 7, but did not differ on days 14 and 21. Feeding a brassica mixture cover crop during the backgrounding phase of production did not impact the collagen content, autolysis of calpain-1, or proteolysis of desmin and troponin-T. Thus, additional investigation into the mechanisms responsible for the differences observed in instrumental tenderness is warranted.

## 1. Introduction

Post-weaning management practices can both positively and negatively impact palatability traits [1,2,3,4]. Supplementation of dietary vitamin D to feedlot steers has been shown to improve the tenderness of strip steaks, as evaluated by both the Warner–Bratzler shear force (WBSF) [1,2] and sensory evaluation [1]. The authors of those studies hypothesized that the additional vitamin D was able to activate increased levels of calpain compared with a control diet [1,2]. Additionally, inclusions of 40% dietary dry matter (DM) of dried distiller’s grains with solubles (DDGS) have also been shown to increase the chance that steaks would be considered as moderately undesirable during retail display compared with diets containing less DDGS [3]. Moreover, supranutritional dietary vitamin E fed to feedlot heifers at the end of finishing was shown to improve the color stability of strip steaks compared with diets without supplemental vitamin E, while injectable trace minerals administered 89 days prior to slaughter increased strip steak discoloration compared with steaks from animals that did not receive injections [4]. 

Little research has been conducted on the backgrounding phase of production, and even less has evaluated the viability of cover crops as a background diet option. Fresh forages such as cover crops are high in vitamins D and E, and could thus influence fresh meat quality characteristics [4,5]. An estimated 6.2 million hectares of cover crops were planted across the United States in 2017 and were used to improve soil health [6]. Brassica cover crops are frequently used in the upper Midwest because of the cold hardiness of kale, forage rape, turnips, and radish [7]. Commonly, these cover crops are used as a low-cost forage for mature cows in order to extend the grazing season. However, the use of cover crops as a feedstuff for weaned calves has been explored by producers, with little research to evaluate the potential impacts on meat quality and palatability. Fehrman [8] observed an improvement in tenderness of strip steaks from steers backgrounded on turnip cover crops compared with steers fed a dry lot diet containing corn stover. However, no other meat quality data were reported. Given the limited amount of research focused on understanding the effects of feeding cover crops in a backgrounding system on beef palatability, the objective of this study was to determine the effects of feeding brassica-based cover crops to steers during backgrounding on the tenderness, juiciness, and flavor of strip steaks. We hypothesize that a brassica mixture cover crop diet during backgrounding would improve tenderness without impacting other palatability attributes. 

## 2. Materials and Methods

### 2.1. Animals and Experimental Diets

**Figure 1:**
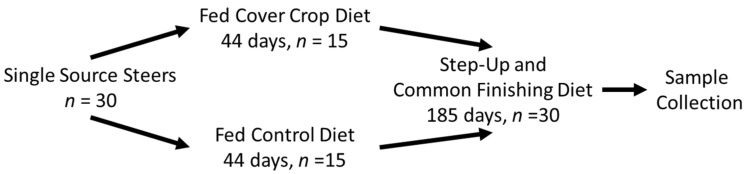
Overall experimental design.

The animal procedures were reviewed and approved by the South Dakota State University Institutional Animal Care and Use Committee (approval number 18-010A). Angus-based steers (*n* = 30; initial body weight 315 ± 25 kg) were obtained from a single local producer. Three days after arrival at the South Dakota State University Cow Calf Education Research Unit (CCERF), the steers were vaccinated for the prevention of Bovine Rhinotracheitis, Parainfluenza 3, Bovine Respiratory Syncytial Virus, *Mannheimia haemolytica*, and Bovine Viral Diarrhea Types 1 and 2 (Inforce™3 and ONE SHOT^®^ BVD, Zoetis Inc., Kalamazoo, MI, USA); administered an anthelmintic (Safe-Guard^®^, Merck Animal Health, Madison, NJ, USA) and an insecticide (Clean-Up™ II; Bayer Healthcare LLC, Shawnee Mission, KS, USA); weighed; and provided an electronic identification tag. The steers were blocked by initial body weight into the light, middle, or heavy blocks, and one of two treatments were assigned randomly to individuals within each block (Figure 1). The steers in the control treatment (CON; initial weight 314 ± 6.6 kg) received a traditional Midwestern backgrounding diet consisting of 54.4% corn silage, 18.8% grass hay, 15% soybean meal, and 11.8% of a commercial liquid supplement (minimum 20% crude protein, 1.85% calcium, 0.2% phosphorus, 4.7% salt, 4% potassium, and 25% moisture) containing 512 g/ton (DM) of monensin on a DM basis. Those of the cover crop treatment (CC) group (initial weight 316 ± 6.6 kg) received a backgrounding diet of freshly cut brassica cover crop foliage, including annual ryegrass (*Lolium multiflorum*; 64.50%), radish (*Raphanus sativus L*.; 15.08%), Trophy rape seed (*Brassica napus*; 9.42%), purple top turnip (*Brassica rapa subsp. rapa*; 9.40%), and the same liquid supplement as the CON treatment. The CC treatment consisted of 95% cover crop and 4.9% liquid supplement on a DM basis. After allocation to treatments, the steers were assigned to individual automated feed bunks that monitored and controlled individual intake (Insentec RIC, Hokofarm Group; Marknessee, Netherlands). Bunk assignments were made based on treatment and initial body weight. The steers were allotted 4 weeks to become acclimated to the feeding system. All of the steers received a common diet of grass hay and corn silage during the acclimation period.

After acclimation was complete, the steers were fed their experimental diets for 44 days, beginning on 15 October 2018. On day 15 of backgrounding, the diets were altered slightly in order to accommodate a change in liquid supplement inclusion. The new CON diet contained 58.1% corn silage, 20.3% grass hay, 16.9% soybean meal, and 4.2% liquid supplement on a DM basis. The CC diet consisted of 96.4% cover crop and 3.6% liquid supplement on a DM basis. The tops of the cover crops were cut daily using a sickle bar mower and were collected using a forage harvester. The cover crops were transported to the CCERF within 1 h of being harvested. Diets were formulated to be isocaloric and isonitrogenous on a DM basis based on feed samples collected prior to study initiation (Table 1). The daily feed intake of each steer was recorded using the feeding system. The steers were paired by body weight and were pair-fed to achieve a similar nutritional profile between treatments. To accomplish a pair feeding system, the steer in the CC treatment was allowed ad libitum access to feed. On the following day, the CON steer of the same pair would have access to the same amount of DM consumed by the CC steer the previous day. Cover crop DM was evaluated weekly, and the diet was adjusted accordingly. Body weights were collected every 7 days for the duration of the backgrounding phase. The backgrounding phase was ended on day 44 as a result of inclement weather that prevented proper harvesting of the cover crop forage.

### 2.2. Finishing Phase, Harvest, and Product Collection

Upon completion of backgrounding, all of the steers were transitioned to a common dry rolled corn-based step up diet including corn earlage, dry rolled corn, DDGS, and liquid supplement containing 512 g/ton (DM basis) of monensin. The step-up diet was conducted over 60 days to a final diet formulated in order to meet or exceed dietary requirements, as outlined by the NRC (Table 1) [9]. The steers were ultrasounded on day 164 for the prediction of slaughter. The endpoint target was when all of the steers reached an average of 1.02 cm of backfat. The steers were transported approximately 240 km to a commercial abattoir for harvest. Strip loins from each carcass, Institutional Meat Purchasing Specification #180, were collected and transported under refrigeration to the South Dakota State University Meat Laboratory for fabrication.

### 2.3. Strip Loin Fabrication

Three days postmortem, the strip loins were trimmed of external fat and the anterior end was removed to obtain an even cut surface prior to slicing 2.54 cm steaks. The anterior portion removed was used for the analysis of the collagen content. The first four steaks were vacuum packaged and aged at 4 °C for 3, 7, 14, or 21 days, respectively, and subsequently frozen at −10 °C to be used for WBSF evaluation. The fifth steak was aged for 14 days and frozen for trained sensory panel evaluation. The eighth steak was quartered and each piece was assigned to age for 3, 7, 14, or 21 days for evaluation of the postmortem proteolysis.

### 2.4. Cook Loss and Warner–Bratzler Shear Force

Steaks designated for WBSF were thawed for 24 h at 4 °C. Prior to cooking, the steaks were weighed (Model MWP, CAS, East Rutherford, NJ, USA) and the initial weight was recorded for cook loss. The teaks were cooked to a target internal temperature of 71 °C using an electric clam shell grill (George Foreman, Model GR2144P, Middleton, WI, USA). The peak internal temperatures were recorded for each steak using a digital thermometer (Atkins Aqua Tuff NSF Series, Cooper-Atkins Corporation, Middlefield, CT, USA). When the steaks were cooled to room temperature, they were weighed again to determine the cook loss (Scale Model MWP, CAS, East Rutherford, NJ, USA). The steaks were stored overnight at 4 °C. The next morning, the steaks were removed from refrigeration and were equilibrated to room temperature before six cores (1.27 cm diameter, 2.54 cm height) were removed parallel to the muscle fiber direction. The cores were sheared perpendicular to the direction of the muscle fibers using a texture analyzer (EZ-SX, Shimadzu Corporation, Kyoto, Japan) with a 50 kg load cell fitted with a Warner–Bratzler shear force head with a crosshead speed of 20 cm/min. The peak force was recorded for each core using texture analyzer manufacture supplied software (Trapezium X, Kyoto, Japan), and the shear force value was determined by averaging the peak force values for all six cores for each steak.

### 2.5. Protein Extraction

The samples used for protein extraction were trimmed of external fat and connective tissue and were prepared by freezing in liquid nitrogen, and then powdered using a Waring commercial blender (Model 51BL32, Waring Products Division, New Hartford, CT, USA) to produce a homogenous sample. The protein samples for gel electrophoresis and Western blot were prepared as described by Melody et al. [10], with several modifications. Briefly, 0.45 to 0.50 g of the sample was weighed and homogenized using an overhead stirrer (model RZR1; Heidolph, Schwabach Germany) in 10 mL of whole muscle buffer (2% sodium dodecyl sulfate [SDS], 10 nM sodium phosphate) to extract the myofibrillar and sarcoplasmic proteins. Homogenized samples were centrifuged for 20 min at 1700× *g* at 25 °C. The protein concentration of the supernatant was determined by diluting in duplicate samples using a 1:20 dilution in double-distilled deionized water. The protein concentrations were determined using a Lowry assay kit (DC Protein Assay Kit; Bio-Rad Laboratories, Hercules, CA, USA) and were analyzed using a spectrophotometer at a wavelength of 750 nm (SpectraMax 190; Molecular Devices, Sunnyvale, CA, USA) and related software (SoftMax Pro 6; version 6.2.1; Molecular Devices, Sunnyvale, CA, USA) that evaluated the protein concentration in relation to a standard curve of bovine serum albumin included in the Lowry assay kit. Gel samples were prepared to a concentration of 4 mg/mL and were stored at −20 °C until analysis [10]. Prior to Western blot analysis, the gel sample protein concentration was confirmed using 15% SDS polyacrylamide separating gels (SDS-PAGE; acrylamide:N,N’-bis-methylene acrylamide = 100:1, 0.1% SDS, 0.05% TEMED, 0.5% ammonium persulfate (APS), and 0.375 M Tris HCl, pH 8.8) with 5% stacking gels (acrylamide:N,N’-bis-methelyene acrylamide = 100:1, 0.1% SDS, 0.125% TEMED, 0.075% APS, and 0.125 M Tris HCl, pH 6.8) to ensure proper dilution of the gel sample. Gels were run using a mini gel electrophoresis unit (model SE-260; Hoefer Scientific, Holliston, MA, USA) at 120 v for 3.25 h. The gels were visualized using a Coomassie blue stain (40% methanol, 7% glacial acetic acid, and 0.1% Coomassie brilliant blue R-250) for 24 h. The gels were destained in 40% methanol and 7% glacial acetic acid. The protein profiles were visually evaluated for similarities across samples (FlourChem M multiflour imaging system; ProteinSimple, Santa Clara, CA, USA) using auto-exposed white light.

### 2.6. Western Blot Analysis

Desmin and troponin-T degradation along with calpain-1 autolysis were determined using 40 µg of the protein sample run on 15%, 15%, and 8% SDS-PAGE gels, respectively, at 120 v for 3 h. Each sample was run in duplicate. After electrophoresis, the gels were transferred to a polyvinylidene difluoride membrane (Immobilon, Darmstadt, Germany) with a pore size of 0.45 µm using a TE-22 transfer unit (Hoefer Scientific, Holliston, MA, USA) for 90 min at 90 v. The transfer buffer (24 mM Tris, 186 mM glycine, and 15% methanol) was maintained at 4 °C using a refrigerated water bath (IsoTemp, model 6200 R28; Thermo Fischer Scientific, Asheville, NC, USA). Once the transfer was complete, membranes were blocked using a 0.5% non-fat dry milk solution for 1 h. Primary antibodies (1:80,000 rabbit anti-desmin (courtesy of the Lonergan Laboratory, Iowa State University, Ames, IA, USA), 1:15,000 monoclonal anti-troponin -T (JLT-12, Sigma, St. Louis, MO, USA), and 1:10,000 monoclonal anti-Mu-Calpain (MA3-940; Thermo Fisher Scientific, Asheville, NC, USA)) were applied to the membranes and were incubated at 4 °C overnight. The blots were equilibrated to room temperature for 1 h prior to being washed with PBS-Tween (66 mM sodium phosphate, 0.1 M NaCl, and 0.1% Tween-20) three times for 10 min each time. A secondary antibody was applied to each membrane (1:20,000 goat anti-rabbit horseradish peroxidase (Thermo Fischer Scientific, Asheville, NC for desmin), 1:20,000 goat anti-mouse horseradish peroxidase for troponin-T, and 1:10,000 goat anti-mouse horseradish peroxidase (Thermo Fisher Scientific, Asheville, NC, USA) for calpain-1) and incubated for 1 h. The blots were washed again three times for 10 min each with PBS-Tween following incubation. The membranes were developed using an ECL Prime detection kit (GE Healthcare, Lafayette, CO, USA). The images were obtained using chemiluminescence with the imaging system previously described. Bands were quantified using AlphaView SA software (ProteinSimple; San Jose, CA, USA). Intact desmin (55 kDa), intact troponin-T (37 kDa), and degraded troponin-T (28 kDa) were analyzed as a ratio to an internal standard. The internal standard was a composite sample equally representing both treatments and all time points used across all Western blots to control for inter gel variation. Calpain-1 autolysis of intact (80 kDa), active (78 kDa), and previously active (76 kDa) calpain-1 was analyzed as a percentage of detected calpain-1 in each sample.

### 2.7. Trained Sensory Panel

Twelve sensory panelists were trained to evaluate the tenderness, juiciness, and beef flavor intensity of strip loin steaks according to the AMSA training guidelines appropriate for the study [11]. The panelists evaluated the attributes on an anchored unmarked continuous 185 mm line scale with the far-left point indicating extremely tender, extremely juicy, or extremely bland beef flavor, and the far-right point representing extremely tough, extremely dry, or extremely intense beef flavor. The steaks were cooked as described for Warner–Bratzler shear force and held at 63 °C in a warming oven (Metro HM2000, Wilkes-Barre, PA, USA). Approximately 15 min prior to sensory evaluation, the steaks were trimmed of external fat and connective tissue and portioned into 2.54 cm × 1 cm × 1 cm cubes. Two cubes were placed into a prelabeled lidded 2 oz plastic cup and returned to the warming oven until they were administered to the panelists.

Evaluations were performed according to AMSA guidelines [11]. Ten samples were evaluated in each session, with one session per day, for a total of three sessions. The sample evaluations were alternated by treatment to reduce first and last order bias. Panelists were secluded by partitioned booths with red lighting and were separated from the steak preparation area. Panelists were provided unsalted crackers and pure water to cleanse their palates between samples.

### 2.8. Collagen Content Analysis

The samples were frozen in liquid nitrogen and were pulverized into finely divided powder. Heat soluble (HS) collagen was extracted from 1-g of powdered, raw meat samples in 5 mL of deionized water at 77 °C for 1 h. The HS collagen extract was cooled on ice, centrifuged at 3000× *g* for 10 min at 4 °C, and separated from the meat pellets (insoluble collagen fraction, IS). Norvaline was introduced into both HS and IS fractions as the internal standard, and either 12N HCl (concentrated, for HS) or 6N HCl (for IS) was added. Acidified HS and IS fractions were placed in a drying oven at 100 ± 2 °C for 16 h. A volume of 50 µL of HS hydrolysate and 5 µL of IS hydrolysates were neutralized with the same volume of 6N NaOH, and were diluted in deionized water to a total volume of 1 mL. The neutralized solutions were centrifuged at 10,000× *g* for 5 min at room temperature. A volume of 200 µL of the neutralized, diluted solutions was reacted with propyl chloroformate in chloroform, sodium hydroxide, and n-propanol, as described by Kaspar et al. [12]. The amino acid derivatives were extracted in isooctane for gas chromatography–mass spectrometry determination. Amino acid derivatives were injected into an inlet of an Agilent 7890A GC System coupled to an Agilent 5975C inert XL MSD with a triple-axis mass detector, an Agilent 7693 Series Autosampler, and a capillary column (Zebron™ EZ-AAA 10 m × 0.25 mm; Phenomenex^®^, Santa Clara, CA, USA). The inlet was operated at 250 °C and had a 1:15 split ratio. The helium carrier gas was at a 1 mL/min constant flow rate. The temperatures of the transfer line, ion source, and quadrupole were 310, 240, and 180 °C, respectively. The oven was programmed initially at 110 °C and ramped up to 320 °C within 11 min. The solvent delay was 1.30 min. The MS was operated in selected ion monitoring (SIM) mode and the target and qualifier ions were selected according to the mass spectra of authentic standards. Amino acids were quantified using an internal calibration method with authentic amino acid standards (Phenomenex^®^, Santa Clara, CA, USA). The collagen content (HS or IS, mg/g) was calculated by multiplying the hydroxyproline concentration with a factor of 7.52 (HS) or 7.25 (IS) [13]. 

### 2.9. Statistical Analysis

All of the data were analyzed as a randomized complete block design using the MIXED procedure of SAS 9.4 (SAS Institute Inc., Cary, NC, USA) with the fixed effect of treatment. Aging day was also used as a fixed effect for WBSF, cook loss, proteolysis, and calpain-1 autolysis. Animal was considered the experimental unit. The cook loss, WBSF, calpain-1 autolysis, and proteolysis data were subjected to different postmortem aging periods and were analyzed as repeated measures using the anti-dependence covariance structure of SAS. Peak internal temperature was used as a covariate for WBSF and cook loss with the Toeplitz covariance structure. Significance was declared at *p* < 0.05. The treatment × aging day interaction was evaluated where appropriate and was reported when significant.

## 3. Results

### 3.1. Warner–Bratzler Shear Force and Cook Loss

**Figure 2:**
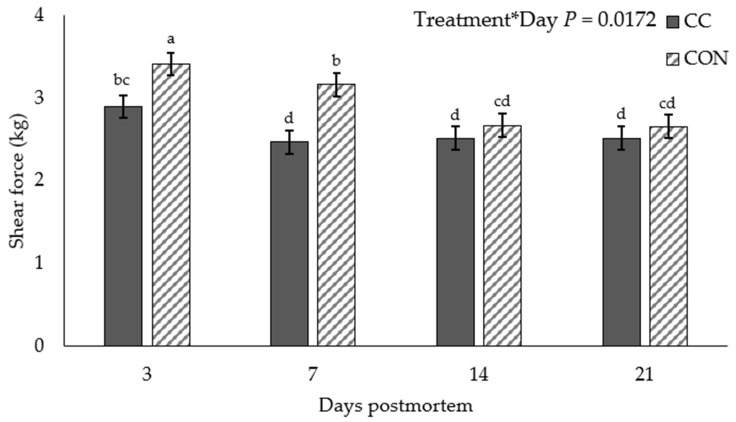
Least squares means for backgrounding treatment: influence on Warner–Bratzler shear force value of strip steaks aged 3, 7, 14, or 21 days postmortem. CC—received a cover crop mixture including brassicas; CON—received a common Midwestern backgrounding diet. ^a b c d^ Means lacking common superscripts differ *p* < 0.05.

A treatment × day interaction was observed for WBSF values (*p* = 0.017; Figure 2). Steaks from the CON treatment group had greater shear force values compared with CC steaks at day 3 (3.41 vs. 2.89 ± 0.14; *p* = 0.011) and 7 (3.16 vs. 2.47 ± 0.14; *p* < 0.001), but were similar on days 14 (*p* = 0.445) and 21 (*p* = 0.477). Additionally, the WBSF values were increased for CON steaks on day 3 compared with days 7 (*p* = 0.045), 14 (*p* < 0.001), and 21 (*p* < 0.001), and the values at day 7 were increased compared with days 14 (*p* < 0.001) and 21 (*p* = 0.001). Steaks in the CC treatment presented increased WBSF values at day 3 compared with days 7 (*p* < 0.007), 14 (*p* = 0.015), and 21 (*p* = 0.044).

Treatment did not impact cook loss (*p* = 0.114; Table 2). However, cook loss increased with the length of postmortem aging (*p* = 0.042; Table 3). Steaks aged for 21 days had greater amounts of cook loss than steaks aged for 3 (*p* = 0.017) or 7 days (*p* = 0.010). 

### 3.2. Western Blot Analysis of Desmin, Troponin-T, and Calpain-1

**Figure 3:**
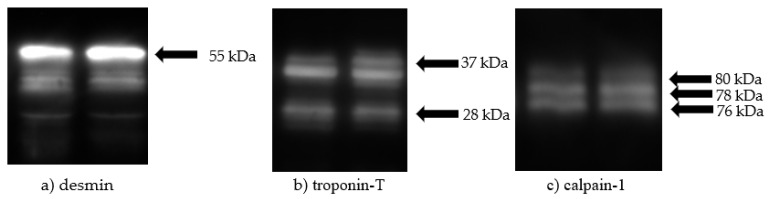
Western blot images of (**a**) intact desmin (55 kDa), (**b**) intact and degraded troponin-T (37 and 28 kDa, respectively), and (**c**) inactive (80 kDa), active, (78 kDa), and previously active (76 kDa) calpain-1.

No treatment effects were observed for the disappearance of intact desmin (55 kDa; Figure 3a) or troponin-T (37 kDa; Figure 3b), or for the appearance of degraded troponin-T (28 kDa; Figure 3b). Further, backgrounding treatment did not influence amount of inactive (80 kDa; Figure 3c) calpain-1, active (78 kDa; Figure 3c) calpain-1, or previously active (76 kDa; Figure 3c) calpain-1 (*p* > 0.05; Table 4). A day effect was observed for desmin, troponin-T, and calpain-1 (*p* < 0.001; Table 3). The abundance of intact desmin decreased from days 3 to 7 (*p* < 0.001), 7 to 14 (*p* < 0.001), and 14 to 21 (*p* = 0.017). The abundance of intact troponin-T was decreased on days 14 and 21 compared with days 3 (*p* < 0.001) and 7 (*p* < 0.001), and abundance decreased for day 7 compared with 3 (*p* < 0.001). Conversely, the abundance of degraded troponin-T increased from days 3 to 7 (*p* = 0.001), 14 (*p* < 0.001), and 21 (*p* < 0.001), as well as from days 7 and 14 to 21 (*p* = 0.013 and *p* = 0.049, respectively).

### 3.3. Sensory Analysis

The trained sensory panel data are presented in Table 2. The tenderness values determined by a trained sensory panel for CC vs. CON steaks did not differ (*p* = 0.346). Additionally, the panelists did not detect differences in juiciness between CC or CON steaks (*p* = 0.428). Moreover, there was no treatment effect on the beef flavor (*p* = 0.204).

### 3.4. Collagen Content

The collagen content is shown in Table 2. Dietary treatment did not impact the amount of insoluble (*p* = 0.111), heat-soluble (*p* = 0.909), and total collagen (*p* = 0.189) in the strip steaks.

## 4. Discussion

The WBSF data suggest steaks from steers backgrounded on brassica cover crops reached ultimate tenderness by day 7, while steaks from the traditional backgrounding diet reached instrumental tenderness by day 14. Similar results were observed by Fehrman [8], as the steaks from steers backgrounded on brassica cover crops were more tender at day 5 compared with the steaks from steers backgrounded on corn stover.

No treatment effects were observed for cook loss. To our knowledge, no other studies have been conducted to evaluate the impact of a backgrounding diet containing brassica cover crops on strip steak cook loss. Cook loss did increase as the aging day increased. Similar results were observed by Shanks et al. [14] when evaluating cook loss over 35-days postmortem. Increases in cook loss over time may be the result of damage to cellular membranes, which would enable a greater amount of moisture to leak out of the muscle during cooking [14].

The Western blot data for desmin, troponin-T, and calpain-1 suggest that while differences in WBSF exist, the proteins and time points selected for evaluation do not explain the differences in tenderness development between treatments. Given that the differences in instrumental tenderness were observed when the experimental diets were fed for only 44 days followed by a 147-day finishing phase, the mechanisms responsible for these differences are likely quite complex. Huff-Lonergan et al. [15] observed an increase in the rate of desmin degradation for steaks with lower WBSF values, and concluded that calpain-1 was likely partially responsible for the degradation. Calpain-1 quickly activates and undergoes autolysis postmortem. Boehm et al. [16] suggested that by 1 day postmortem, calpain-1 has only 20% of its at-death activity. Therefore, the conflicting data for WBSF and proteolysis in the current study were possibly due to a faster rate of calpain autolysis prior to day 3 sampling for the CON treatment group compared with the CC treatment group. Troponin-T is categorized as a regulatory protein that regulates contraction. Troponin-T is used as an indicator of tenderness as it provides little structural support, and its breakdown will not cause the physical disruption of myofibers needed for tenderness development [17,18]. Although, as troponin-T degradation has been established as a good indicator of tenderness development, it is unclear why intact and degraded troponin-T did not differ in the current study where WBSF differences exist. However, while not statistically significant, the proteolysis data for desmin and troponin-T numerically support the WBSF data. The data from the CC treatment indicate numeric decreases in intact desmin and troponin-T, and there is a numeric increase in degraded troponin-T compared with the CON treatment. Thus, it is possible that an additive effect of proteolysis for the proteins evaluated can partially explain the observed differences in WBSF. The Western blot data for aging day effect of proteolysis of desmin and troponin-T and calpain-1 autolysis are not unexpected, as it is well documented that myofibrillar proteins degrade during postmortem storage [15,16,19].

Trained sensory panelists did not detect differences in the tenderness, juiciness, or flavor of strip steaks between each treatment. Notably, the panel results support the day 14 WBSF and cook loss results as no differences were observed for those data. Thus, differences in tenderness and juiciness were not expected. Similarly, Duckett et al. [20] evaluated the impact of winter stocker growth on palatability and observed no differences in flavor by a trained panel. Therefore, it is likely that any impact backgrounding diet had on flavor was dissipated via the common finishing period.

Traditionally, the collagen content has been regarded as being responsible for background toughness, which cannot be reversed by postmortem aging [21]. Calkins and Sullivan [22] indicated that the overall impact of connective tissue on tenderness is influenced by both the amount of connective tissue present and its solubility. While none of the collagen data are statistically significant, the tendency for the abundance of insoluble collagen does reflect the WBSF data, as steaks from the CC treatment group had lower values than the steaks from the CON treatment. Previous research has indicated that ruminant diets containing high levels of vitamins E and C (both commonly found in fresh forages) can positively impact tenderness [23]. An in vivo study found that fibroblasts subjected to a combination of vitamins E and C activated matrix metalloproteinase-2 (MMP-2), which has been shown to degrade collagen [23]. The activation of MMP-2 could increase protein turnover by triggering the degradation of older collagen and the production of immature collagen, which is more soluble [23]. However, additional work must be conducted to establish whether similar results could be replicated in a conventional beef production system.

## 5. Conclusions

The results of this study show that dietary management, including using brassicas during the backgrounding phase, as well as the aging day, may influence instrumental tenderness, even after a common finishing diet. Tenderness as measured by WBSF was improved more rapidly in steaks from the CC treatment, but differences were not detected after 14 days of postmortem aging, and no treatment effects were observed for proteolysis or sensory data. Additional research is warranted in order continue to evaluate the impacts of dietary brassica cover crop forages during backgrounding on meat quality.

## Figures and Tables

**Table 1:** Backgrounding and finishing diet composition for steers backgrounded on a cover crop mixture including brassicas (CC) or a common Midwestern backgrounding diet (CON) ^1^.

Nutrient Composition ^2^	CC	CON	CC	CON	Finishing
	day 0–14		day 15–44		day 106–231
Crude Protein	13.31	17.02	13.06	16.10	13.73
NE_m_, Mcal/cwt ^3^	60.15	71.62	60.11	72.35	86.03
NE_g_, Mcal/cwt ^4^	34.34	44.93	34.25	45.26	58.14

^1^ CC = received a cover crop mixture including brassicas, CON = received a common midwestern backgrounding diet. ^2^ Calculated on a dry matter basis. ^3^ Net energy, maintenance. ^4^ Net energy, gain.

**Table 2:** Least square means for the main effect of the background treatment influence ^1^ on strip steak cook loss, trained sensory panel attributes, and collagen content.

Variable	CC	CON	SEM ^2^	*p*-Value
Cook loss, %	17.56	18.78	0.528	0.114
Tenderness ^3^	68.24	74.00	4.389	0.346
Juiciness ^3^	97.28	92.22	4.552	0.428
Flavor ^3^	80.85	86.16	3.054	0.204
Insoluble collagen, mg/g	1.95	2.02	0.041	0.111
Heat soluble collagen, mg/g	0.25	0.24	0.015	0.909
Total collagen, mg/g	2.20	2.26	0.049	0.189

^1^ CC—received a cover crop mixture including brassicas; CON—received a common Midwestern backgrounding diet. ^2^ Standard error of means. ^3^ Evaluated on an anchored unmarked 185 mm line scale where 0 indicates extremely tender, extremely juicy, or extremely bland beef flavor, and 185 indicates extremely tough, extremely dry, or extremely intense beef flavor.

**Table 3:** Least square means of the impact of aging day on cook loss, proteolysis of desmin and troponin-T, and autolysis of calpain-1 of strip steaks from steers fed CC and CON diets ^1^ during backgrounding.

Variable	3	7	14	21	21	SEM ^2^	*p*-Value
Cook loss, %	17.86 ^b^	17.27 ^b^	18.21 ^ab^	19.36 ^a^	19.36 ^a^	0.579	0.042
Intact desmin (55 kDa) ^3^	1.4994 ^a^	1.0025 ^b^	0.7607 ^c^	0.6053 ^d^	0.6053 ^d^	0.064	<0.001
Intact troponin-T (37 kDa) ^3^	1.3437 ^a^	0.9993 ^b^	0.6769 ^c^	0.5887 ^c^	0.5887 ^c^	0.045	<0.001
Degraded troponin-T (28 kDa) ^3^	0.6758 ^c^	1.0216 ^b^	1.0786 ^b^	1.2881 ^a^	1.2881 ^a^	0.074	<0.001
Inactive calpain-1 (80 kDa) ^4^, %	13.46	7.45	-	-	-	0.899	<0.001
Active calpain-1 (78 kDa) ^4^, %	34.21	30.03	-	-	-	0.735	<0.001
Previously active calpain-1 (76 kDa) ^4^, %	52.22	62.47	-	-	-	1.373	<0.001

^1^ CC—received a cover crop mixture including brassicas; CON—received a common Midwestern backgrounding diet. ^2^ Standard error of means. ^3^ Expressed as a ratio to an internal standard. ^4^ Expressed as a percentage of total calpain-1. ^a b c d^ Means lacking common superscripts differ *p* < 0.05.

**Table 4:** Least square means for the main effect of background treatment influence ^1^ on strip steak proteolysis of desmin and troponin-T, and autolysis of calpain-1.

Variable	CC	CON	SEM ^2^	*p*-Value
Intact desmin (55 kDa) ^3^	0.9262	1.0076	0.047	0.215
Intact troponin-T (37 kDa) ^3^	0.8926	0.9117	0.032	0.673
Degraded troponin-T (28 kDa) ^3^	1.0589	0.9781	0.052	0.314
Inactive calpain-1 (80 kDa) ^4^, %	9.99	10.92	0.899	0.470
Active calpain-1 (78 kDa) ^4^, %	31.71	32.52	0.735	0.444
Previously active calpain-1 (76 kDa) ^4^, %	58.24	56.44	1.373	0.361

^1^ CC—received a cover crop mixture including brassicas; CON—received a common Midwestern backgrounding diet. ^2^ Standard error of means. ^3^ Expressed as a ratio to an internal standard. ^4^ Expressed as a percentage of total calpain-1.

## Data Availability

The data presented in this study are available upon request from the corresponding author.
